# WRN Loss Induces Switching of Telomerase-Independent Mechanisms of Telomere Elongation

**DOI:** 10.1371/journal.pone.0093991

**Published:** 2014-04-07

**Authors:** April Renee Sandy Gocha, Samir Acharya, Joanna Groden

**Affiliations:** Department of Molecular Virology, Immunology and Medical Genetics, The Ohio State University College of Medicine, Columbus, Ohio, United States of America; The University of Hong Kong, Hong Kong

## Abstract

Telomere maintenance can occur in the presence of telomerase or in its absence, termed alternative lengthening of telomeres (ALT). ALT adds telomere repeats using recombination-based processes and DNA repair proteins that function in homologous recombination. Our previous work reported that the RecQ-like BLM helicase is required for ALT and that it unwinds telomeric substrates *in vitro*. WRN is also a RecQ-like helicase that shares many biochemical functions with BLM. WRN interacts with BLM, unwinds telomeric substrates, and co-localizes to ALT-associated PML bodies (APBs), suggesting that it may also be required for ALT processes. Using long-term *siRNA* knockdown of WRN in three ALT cell lines, we show that some, but not all, cell lines require WRN for telomere maintenance. VA-13 cells require WRN to prevent telomere loss and for the formation of APBs; Saos-2 cells do not. A third ALT cell line, U-2 OS, requires WRN for APB formation, however WRN loss results in p53-mediated apoptosis. In the absence of WRN and p53, U-2 OS cells undergo telomere loss for an intermediate number of population doublings (50–70), at which point they maintain telomere length even with the continued loss of WRN. WRN and the tumor suppressor BRCA1 co-localize to APBs in VA-13 and U-2 OS, but not in Saos-2 cells. WRN loss in U-2 OS is associated with a loss of BRCA1 from APBs. While the loss of WRN significantly increases telomere sister chromatid exchanges (T-SCE) in these three ALT cell lines, loss of both BRCA1 and WRN does not significantly alter T-SCE. This work demonstrates that ALT cell lines use different telomerase-independent maintenance mechanisms that variably require the WRN helicase and that some cells can switch from one mechanism to another that permits telomere elongation in the absence of WRN. Our data suggest that BRCA1 localization may define these mechanisms.

## Introduction

Shortening of chromosome ends persists in normal cells with each round of cell division due to inability of the replication machinery to proceed completely to the chromosome end. Telomeres protect the terminal chromosome ends by buffering the loss of coding DNA and by concealing the chromosome end from recognition as a DNA double strand break. Although most human somatic cells do not maintain their telomeres, cancer cells activate a telomere maintenance mechanism to support growth and immortalization. A majority of cancers activate telomerase, while a subset maintains telomeres in the absence of telomerase. This telomere maintenance mechanism is termed alternative lengthening of telomeres, or ALT.

Yeast cells can survive without telomerase in two RAD52-dependent forms [1]: type I survivors are RAD51-dependent and have short telomere repeats and amplified Y′ telomere elements, while type II yeast are RAD50-dependent and have long heterogeneous telomere repeat tracts. Immortalized mammalian cells without detectable telomerase expression are classified as ALT. ALT characteristics include heterogeneous telomere lengths, extrachromosomal telomeric repeats (ECTR) and ALT-associated PML bodies (APBs), although these characteristics are variable [2], [3]. Evidence suggests that ALT uses recombination to add telomeric repeats to the chromosome terminus [4]; homologous or non-homologous chromosome ends can supply a (TTAGGG)_n_ template for telomeric recombination. Alternatively, sister chromatids or extra-chromosomal telomere repeats (ECTR), abundant in both linear and circular forms in ALT cells, may provide templates for telomere elongation by *de novo* addition (linear) or rolling circle amplification (circular). Both inter-telomeric copying between chromosomes [4] and intra-telomeric copying within the same chromosome [5] have been shown. Given these options, multiple telomerase-independent maintenance mechanisms may be used by mammalian cells, reminiscent of the yeast type I and II pathways.

Numerous DNA repair proteins localize to the telomere in ALT cells and are assimilated in APBs. APBs are distinguished from normal PML bodies by the inclusion of telomeric proteins and telomere DNA [6] and are sites of bromo-deoxyuridine (BrdU) incorporation [7]. APBs are considered sites of telomere elongation and/or dynamics in ALT cells. DNA repair proteins, such as the MRN complex [7], localize to APBs and may function to recognize or protect telomeric intermediates in ALT recombination processes or may actively participate in telomeric recombination. Two other DNA repair proteins, the RecQ-like BLM [8] and WRN [9] helicases localize to APBs.

Bloom syndrome (BS) and Werner syndrome (WS) are inherited chromosome instability disorders marked by predisposition to cancer. The genes mutated in BS and WS, *BLM* and *WRN*, respectively, encode proteins of similar structure that carry out essential roles in DNA replication and repair. Both helicases unwind telomeric DNA sequences and structures *in vitro* [10], [11], suggesting that they unwind the terminal telomere loop structure (T-loop) to allow telomere elongation. BLM [8] and WRN [11] interact with telomeric proteins and telomere DNA in immortalized human cell lines. The telomeric proteins TRF2 [10], [12], [13] and POT1 [14] interact with BLM and WRN to stimulate helicase activity using telomeric substrates *in vitro*.

Evidence suggests that telomere dysfunction contributes to disease pathology in both BS and WS: BS cells exhibit telomeric associations [10], while cultured WS cells undergo early senescence [15], [16] and exhibit increased telomeric sister chromatid exchange [17] and telomere loss [18]. Exogenous telomerase expression prevents early senescence and telomere loss in WS cells [18], [19], indicating shortened telomeres may contribute to disease progression. *Wrn*-deficient mice lack the WS disease phenotype [20], although late generation mice develop phenotypes that closely mimic WS when *Wrn* is mutated in combination with *Terc*, the RNA component of telomerase [21]. Normal mice display these phenotypes only in the context of shortened telomeres, suggesting that the *Wrn^−/−^* manifestations are associated with shortened telomeres. Mutation of *Blm, Wrn* and *Terc* accentuates telomere dysfunction, suggesting that all proteins function at the telomere [22].

Our previous work showed that BLM is required to maintain telomeres in ALT cells, but not in telomerase-positive cells [23]. The similarities in structure and *in vitro* function of BLM and WRN suggested that WRN may also be required for ALT. A previous study concluded that WRN is dispensable for ALT based upon a WS cell line that displays ALT characteristics, AG11395 [2]. AG11395 is SV40-immortalized, incorporates SV40 sequences into its telomeric repeats and fails to form APBs. Expression of WRN in AG11395 cells results in APB formation, gain of telomeric sequences and the conversion of type I-like telomeres (with SV40 sequences interspersed) to type II-like telomeres [24] and suggests that WRN can function at the telomere in some ALT mechanisms. Therefore, we asked whether WRN is required for ALT by investigating its role in three other ALT cell lines. Our results show that two of these three ALT cell lines require WRN to maintain telomeres by preventing telomere loss and promoting APB formation. In addition, the requirement for WRN in ALT is associated with an ALT-specific interaction between WRN and the BRCA1 tumor suppressor. Finally, we present evidence for an ALT mechanism switch in cells undergoing long-term *siRNA* knockdown of *WRN* and *p53* confirming that ALT cells can use different pathways of telomerase-independent telomere maintenance, that these pathways employ unique subsets of proteins, and that cells have the ability to switch ALT mechanisms when telomeres begin to shorten.

## Results

### Continuous WRN knockdown shortens telomeres in VA-13 and U-2 OS, but not Saos-2, ALT cell lines

**Figure 1:**
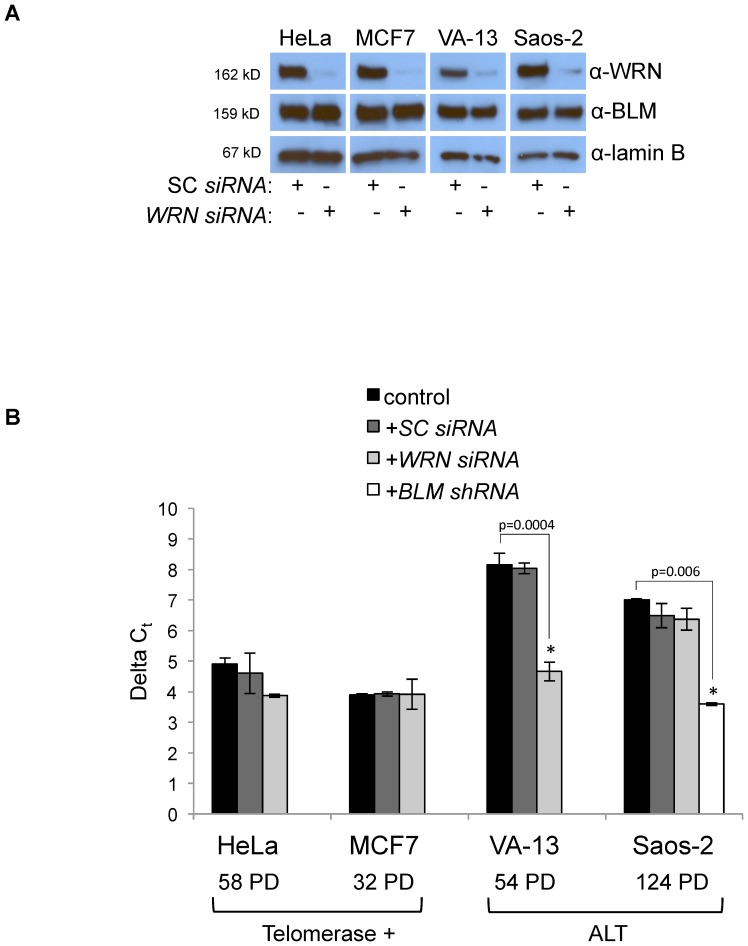
*WRN siRNA* knockdown and telomere length of HeLa, MCF7, VA-13, and Saos-2 cells. **A**. Pooled *WRN siRNAs* or scrambled control (SC) *siRNAs* were transfected into immortalized human cell lines and whole cell extracts were collected 48 hours after transfection. Each lysate was separated by SDS-PAGE and western blotted with antibodies to WRN (top), BLM (to ensure specificity of the *siRNAs,* middle), and lamin B (as a loading control, bottom). **B**. Relative telomere length measurement by qRT-PCR. The difference in the cycle threshold (C_t_) between a telomere-specific PCR reaction and a single copy gene (*ALB*) PCR reaction is calculated for each sample as the ΔC_t_, which represents the average relative telomere repeat length. Telomere length in Saos-2 ALT cells decreases after stable *BLM* knockdown as measured by TRF Southern blot [23]. Here, we confirm detection of these length changes by qRT-PCR (far right). Telomere lengths of telomerase-positive cells, HeLa and MCF7, and ALT cells, VA-13 and Saos2, were measured following transfection of either SC or *WRN siRNAs*. Relative lengths depicted represent the final measurement for each cell type, which was taken at the population doubling number (PD) indicated. (ALT = alternative lengthening of telomeres; TRF =  telomere restriction fragment; PD = population doubling).

Telomeres shorten an average of 50–100 bp per cell division in the absence of a telomere maintenance mechanism [25]–[28]. To investigate whether WRN is necessary to maintain telomeres in the absence of telomerase, we continuously knocked down *WRN* in two telomerase-positive immortalized human cell lines, HeLa and MCF7, and two ALT immortalized human cell lines, WI-38 VA-13/2RA (hereafter referred to as VA-13) and Saos-2. Attempts to generate stable clones using two pSilencer*WRN shRNA* plasmids generously provided by Dr. Patricia Opresko were unsuccessful. Although multiple antibiotic-resistant cell clones were generated, WRN continued to be expressed as assessed by western blot (data not shown). As an alternative strategy, pooled *WRN siRNAs* were reverse-transfected into cells every five days to maintain a continuous transient knockdown. Cells were maintained in culture for at least 30 population doublings (PD) with continuous transient *WRN* knockdown to allow changes in telomere length to occur. PD times ranged from about 24 hours for HeLa and U-2 OS cells to almost 48 hours for MCF7 cells. Although PD differed slightly among cell lines, cell cycle distribution and progression were similar. WRN expression was consistently reduced by over 90% in all cell lines (**Fig 1A**, top panels) as compared to the loading control lamin B (**Fig 1A**, bottom panels). Figure 1 depicts a representative knockdown of WRN examined at 48 hours after transfection; western blots showed similar reductions five days after initial transfection (**Fig S1**) and throughout the entire course of the experiment. *BLM* and *WRN* are highly homologous; therefore western blotting verified that *WRN siRNAs* were specific to *WRN* and did not affect *BLM* expression levels (**Fig 1A**, middle panels). Cells were transfected with pooled scrambled control (SC) *siRNAs* as a negative control. No growth defects were observed in cells transfected with *WRN siRNAs* compared to those transfected with SC *siRNAs* or untransfected cells. Cells were then analyzed for characteristics of telomere maintenance mechanisms.

To examine the requirement for WRN in telomere maintenance, relative telomere lengths were measured in cells maintained with continuous *WRN* knockdown. Genomic DNA was extracted from cells at 4, 20 and 54 population doublings (PD) after initiation of *WRN* knockdown and telomere repeat length analyzed by quantitative real-time PCR (qRT-PCR) [29]. qRT-PCR allows a quantitative relative telomere length measurement from a small amount of DNA, in comparison to telomere restriction fragment Southern blotting (TRF Southern); because we performed long-term *siRNA* knockdowns in small cell populations, sample DNA amounts only permitted qRT-PCR measurement of telomeres. However, to validate the ability of qRT-PCR to measure telomere length accurately, we performed both qRT-PCR and telomere restriction fragment (TRF) Southern blotting on a separate subset of DNA samples, which showed good correlation between the two methods (**Fig S2**). In addition, we confirmed the ability of qRT-PCR to measure reported changes in telomere length after stable *BLM* knockdown in Saos-2 cells, previously measured by TRF Southern blot (**Fig 1B**) [23]. A significant reduction in relative mean telomere length after *BLM* knockdown (p = 0.006) was observed. Relative telomere length in two telomerase-positive cell lines was unchanged in response to continuous transfection with SC (HeLa *p* = 0.506; MCF7 *p* = 0.415) or *WRN siRNAs* (HeLa *p* = 0.195; MCF7 *p* = 0.987) at all PDs sampled (**Fig 1B**). HeLa cells were maintained in culture for 58 PD and MCF7 cells were maintained for 32 PD. In contrast, VA-13 ALT cells were unable to maintain telomere lengths without WRN. Telomere lengths remained stable for 20 PD, but then steadily shortened to almost 50% of their original length by 54 PD (*p* = 0.0004). Remaining cells in culture declined in health until no cells survived at 66 PD, presumably due to progressively shortening telomeres; sufficient cell numbers for analysis could not be collected after 54 PD. No significant change in telomere length was observed in untransfected controls or SC *siRNA* transfected VA-13 cells (*p* = 0.670), indicating the telomere length reductions were a specific response to *WRN* knockdown (**Fig 1B**). In contrast, Saos-2 ALT cells steadily maintained long telomere lengths despite high *WRN* knockdown efficiency (**Fig 1A, 1B**). Saos-2 cells were maintained in culture for 124 PD without a significant change in telomere length in response to *WRN siRNAs* (*p* = 0.732) or SC *siRNA*s (*p* = 0.146). These results suggest that VA-13 cells require WRN for alternative telomere maintenance and that Saos-2 cells do not.

**Figure 2:**
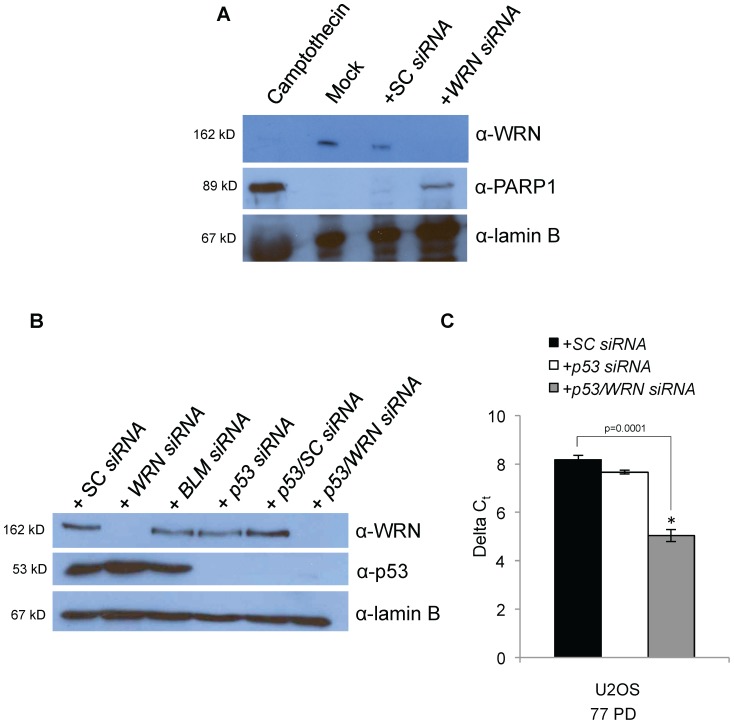
*WRN siRNA* knockdown and telomere length of U-2 OS cells. **A**. U-2 OS cells were mock transfected or transfected with SC or *WRN siRNAs*. Whole cell extracts were collected, separated by SDS-PAGE and western blotted with antibodies to WRN to confirm the knockdown (top panel), cleaved PARP1 to examine apoptosis induction (middle panel) and lamin B as a loading control (bottom panel). Treatment with camptothecin is a positive control for the induction of apoptosis. **B**. To inhibit apoptosis, U-2 OS cells were transfected with *WRN* and *p53 siRNAs*. Whole cell extracts were separated by SDS-PAGE and western blotted with antibodies specific to WRN (top panel), p53 (middle panel) and lamin B (loading control; bottom panel). **C**. DNA from transfected U-2 OS cells was collected at 77 PD and telomere length was measured by qRT-PCR. Average relative telomere repeat lengths are graphically represented as the difference between a telomere-specific PCR reaction in comparison to the single copy gene *ALB* PCR reaction, or delta C_t_. (ALT = alternative lengthening of telomeres; PD = population doubling)

We asked whether our results were unique to VA-13 cells and transfected the U-2 OS ALT cell line with *WRN siRNAs*. U-2 OS ALT cells are established from an osteosarcoma but maintain an intact p53 pathway although most other ALT cell lines, including VA-13 and Saos-2, are mutated or null for *p53*. U-2 OS cells died in culture in response to *WRN* knockdown after 17 PD accompanied by an increase in cleaved poly-ADP ribose polymerase 1 (PARP1) (∼30% induction compared to camptothecin treatment) following transfection with *WRN siRNAs* (**Fig 2A**). No change in telomere length was detected during this time, indicating that U-2 OS cells without WRN undergo apoptosis before a change in telomere length is detected. To test whether WRN is required for ALT in U-2 OS cells, U-2 OS cells were simultaneously transfected with *siRNAs* to reduce both *WRN* and *p53*; western blots demonstrated that both proteins were effectively reduced (**Fig 2B**). Cells were maintained in culture with continuous transient knockdown as described previously. A significant reduction in telomere length (38%) was detected at 77 PD in U-2 OS cells transfected with *WRN* and *p53 siRNAs* (**Fig 2C**).

The ability of a cell or tumor to switch telomere maintenance mechanism is not well understood, although recent publications suggest that this may occur [30]. As Ballal *et al.* [31] reported that telomerase expression increased in cell cultures following *BRCA1* knockdown, the telomere repeat amplification protocol (TRAP) assay was used to assess telomerase activity in cell extracts of Saos-2 ALT cells to test whether telomerase reactivation permitted their ability to maintain telomeres without WRN. Non-transfected Saos-2 cells do not express telomerase and exhibited no activity in the TRAP assay; transfections with SC or *WRN siRNAs* did not result in telomerase activation (**Fig S3**). Positive controls for the TRAP assay included extracts from telomerase-positive MCF7 and HeLa cells. Activity was absent in VA-13 and U-2 OS ALT cell lines (**Fig S3**). These results support the conclusion that WRN is not required for ALT in Saos-2 cells.

### Loss of WRN prevents APB formation in VA-13 and U-2 OS cells

**Figure 3:**
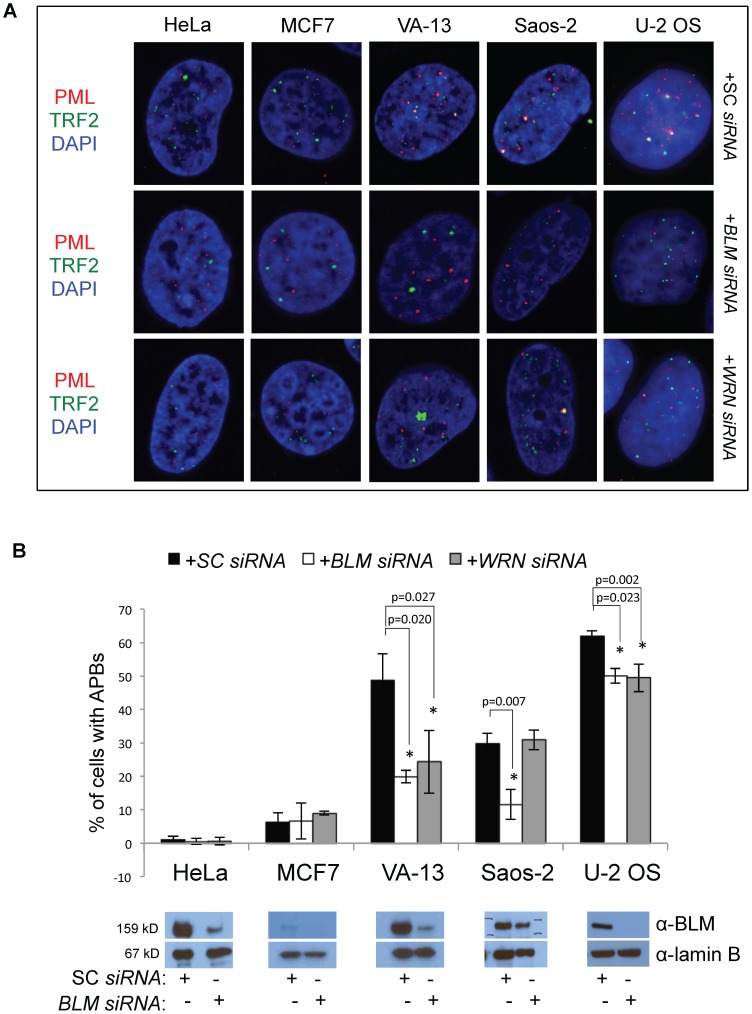
ALT-associated PML body (APB) formation following *WRN* knockdown. **A**. Representative confocal micrograph images of cells from two telomerase-positive cell lines, HeLa and MCF7, and three ALT cell lines, VA-13, Saos-2 and U-2 OS. Cells were fixed 48 hours after transfection with scrambled control (SC) *siRNAs*, *BLM siRNAs* or *WRN siRNAs* and immunofluorescently labeled with antibodies to PML and TRF2. We have previously shown a reduction in APBs following *BLM* knockdown [23], so cells transfected with BLM *siRNAs* served as a positive control. PML is labeled in red, TRF2 in green, and nuclei stained with DAPI (blue). **B**. A graphical representation of at least 3 independent experiments depicts the percentage of cells displaying co-localization of TRF2 and PML, indicative of APBs. Co-localization of PML/TRF2 foci was scored as a positive indication of APBs; at least three independent experiments were averaged to yield the percentage of each cell type. Western blots confirm the ability of *BLM siRNAs* to reduce BLM expression.

ALT characteristics of VA-13 and U-2 OS cells were analyzed following *WRN* knockdown. APBs, in which PML co-localizes with the telomeric protein TRF2, are characteristic of ALT cells and are quickly responsive to changes in ALT activity [32]–[34], suggesting that these nuclear bodies reflect the type of telomere maintenance mechanism used by cells. APBs were visualized in fixed cells by immunofluorescence with antibodies to TRF2 and PML following *WRN* or SC *siRNA* transfection (**Fig 3A**). APBs sharply decline in ALT cells upon *BLM* knockdown [23], so these cell lines were also transfected with *BLM siRNAs* to provide a positive control. Control VA-13 (48.7% of cells), Saos-2 (29.8% of cells) and U-2 OS (61.9% of cells) cells displayed significant co-localization of TRF2 with PML, while both telomerase-positive cell lines lacked APBs (**Fig 3B**). Forty-eight hours following *WRN* knockdown, APB formation was reduced by 50.1% in VA-13 cells (*p* = 0.027) and by 20.0% in U-2 OS cells (*p* = 0.023). Saos-2 cells displayed no reduction in APBs (*p* = 0.698); telomerase-positive cells also displayed no change (HeLa *p* = 0.613; MCF7 *p* = 0.211). *BLM* knockdown significantly reduced APB levels in all ALT cell lines (59.1% reduction in VA-13, *p* = 0.020; 19.1% reduction in U-2 OS, *p* = 0.002; and 61.1% reduction in Saos-2, *p* = 0.007) and had no effect on the telomerase-positive cell lines (HeLa, *p* = 0.502; MCF7, *p* = 0.937). These results implicate a function for WRN in APB formation in cells that require WRN for ALT.

ECTR are abundant in ALT cells and are most commonly present in a partially single-stranded, C-rich form termed C-circles [35]. C-circles are a predictor of ALT activity and can be measured *in vitro* with an assay that promotes rolling circle amplification of C-circles to produce a large and abundant telomeric product [35]. We used the C-circle assay to examine ECTR in VA-13 and U-2 OS cells after transfection with SC or *WRN siRNAs* and found no differences in the prevalence of C-circles before or after WRN knockdown (**Fig S4**). This result held true for multiple time points after continuous *WRN siRNA* knockdown, including 4, 20 and 54 PD. The C-circle status of all cell lines tested was unaffected by *siRNA* transfection, suggesting that loss of WRN does not influence the prevalence of C-circles in the cells tested. These results are consistent with those obtained in CCL75.1 ALT cells, in which *WRN* knockdown does not alter the levels of T-circles [36].

### WRN is required for ALT telomere maintenance in VA-13 cells

**Figure 4:**
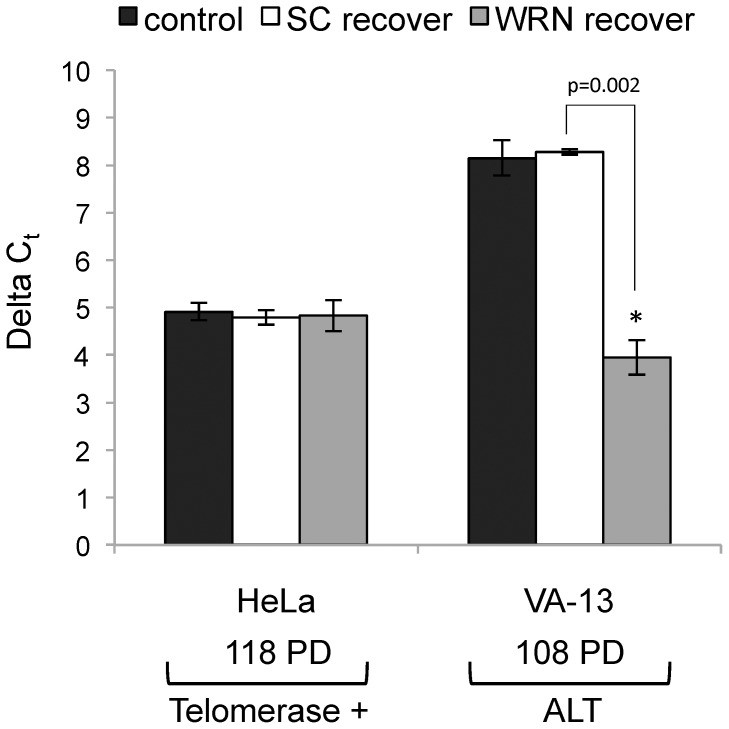
Telomere length after recovery from *WRN siRNA* transfection. After 54 PD of continuous *WRN* knockdown (after telomeres had shortened by almost 50% of the original length), *WRN siRNA* transfections of a subset of HeLa and VA-13 cells were ceased and cells allowed to recover in culture for an additional 54 PD. Average relative telomere repeat lengths were then measured by qRT-PCR. Results are graphically represented as the difference between a telomere-specific PCR reaction in comparison to the single copy gene albumin PCR reaction as delta C_t_.

Telomere lengths drastically shorten after prolonged *WRN* knockdown in VA-13 cells, eventually resulting in cell death at 66 PD. We asked if re-expression of endogenous WRN could reverse this telomere loss. A 50% reduction in telomere length was observed at 54 PD of continuous *WRN* knockdown. A subset of VA-13 cells was separated and grown in culture for an additional 54 PD without further *siRNA* transfection. A sample of non-transfected control VA-13 cells and a sample transfected with SC *siRNA*s for 54 PD were also cultured without further *siRNA* transfection. Samples of control HeLa cells and those transfected with SC and *WRN siRNAs* were cultured without further *siRNA* transfections as a negative control. Telomere length in VA-13 cells stabilized at the shortened 50% length and remained steady for an additional 54 PD in the absence of *WRN* knockdown (total of 108 PD) (**Fig 4**). Telomere lengths did not change in control VA-13 cells or after recovery from SC *siRNA* (*p* = 0.622) and no significant change was observed in HeLa cells. Although WRN was re-expressed in cells at levels comparable to untransfected cells (data not shown), the VA-13 telomeres were unable to elongate to the same length as non-transfected control cells (*p* = 0.002), but were elongated to maintain the new, shorter length in recovered cells. These results suggest that WRN is necessary to maintain telomeres in VA-13 ALT cells.

### WRN does not cap telomere ends

**Figure 5:**
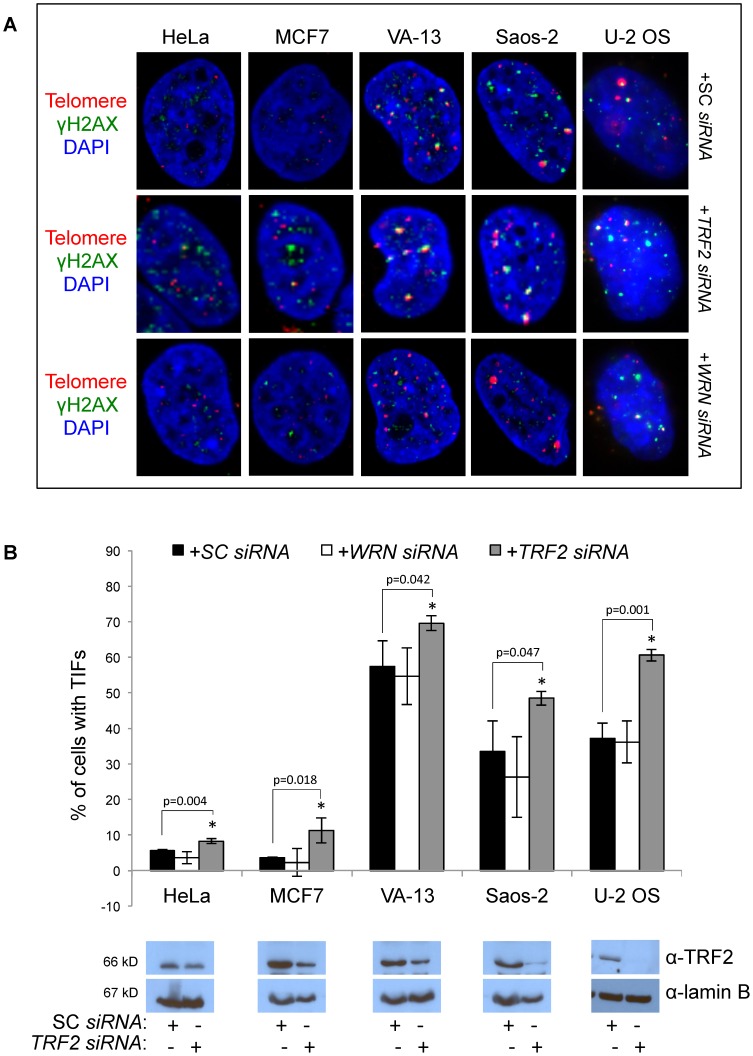
Telomere dysfunction-induced foci (TIF) formation after *WRN* knockdown. **A**. Representative confocal micrograph images of cells from two telomerase positive cell lines, HeLa and MCF7, and three ALT cell lines, VA-13, Saos-2 and U-2 OS. Cells were fixed 48 hours after transfection with scrambled control (SC) *siRNAs*, *WRN siRNAs* or *TRF2 siRNAs* and subjected to fluorescent *in situ* hybridization (FISH) with a Cy3-labeled telomeric PNA probe and immunofluorescently labeled with an antibody to phosphorylated histone 2A (γH2AX). As TRF2 is a member of the shelterin complex, *TRF2* knockdown was used as a positive control. The telomere is labeled with a Cy3-PNA probe in red, γH2AX in green, and the nucleus is stained with DAPI (blue). **B**. A graphical representation of at least three independent experiments were averaged to yield the percentage of each cell type with γH2AX co-localization with the telomere, indicative of telomere dysfunction induced foci (TIF). Western blots confirm the ability of *TRF2 siRNAs* to reduce TRF2 expression.

The shelterin protein complex—composed of TRF1, TRF2, TIN2, TPP1, RAP1 and POT1—caps the telomere and hides the chromosome end from recognition as a DNA break. Loss of shelterin components results in a robust DNA damage response at the telomere and telomere dysfunction [37]–[40]. Telomere dysfunction-induced foci (TIFs) form at telomeres in response to DNA damage and are visualized by co-localization of DNA damage response proteins, including γH2AX and 53BP1, with the telomere. ALT cells maintain basal levels of telomere dysfunction [41] and display TIFs in the absence of DNA damaging treatment. We tested whether WRN may act as a telomere capping protein as *WRN* knockdown resulted in telomere loss in VA-13 and U-2 OS ALT cells and restoration of WRN expression following telomere shortening in VA-13 cells failed to return telomeres to original lengths. Co-localization of γH2AX with the telomere was immunofluorescently analyzed in fixed control cells and those transfected with SC, *WRN* or *TRF2 siRNAs* (**Fig 5A**). *TRF2* knockdown served as a positive control for the induction of TIFs and resulted in a significant uncapping of telomeres in all cell lines tested (HeLa *p* = 0.004; MCF7 *p* = 0.018; VA-13 *p* = 0.047; Saos-2 *p* = 0.042; U-2 OS *p* = 0.001), although TRF2 expression was only modestly knocked down by western blot. *WRN* knockdown did not alter TIFs in any cell line tested (HeLa *p* = 0.124; MCF7 *p* = 0.597; VA-13 *p* = 0.742; Saos-2 *p* = 0.345; U-2 OS *p* = 0.819) (**Fig 5B**), suggesting that WRN does not function to cap telomere ends.

### Differential requirement for WRN in ALT reflects differences in WRN protein interactions

**Figure 6:**
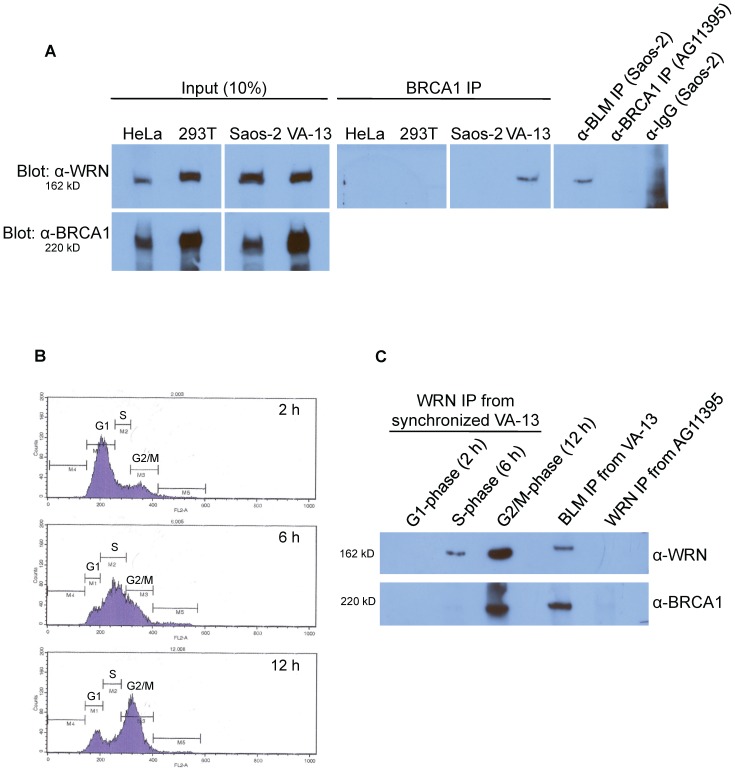
WRN immunoprecipitation from telomerase-positive and ALT cells. **A**. Nuclear extracts were immunoprecipitated with antibodies to BRCA1 and the immunoprecipitated proteins subjected to western blotting with an antibody to WRN (right panels). Immunoprecipitation with anti-BLM serves as a positive control for the interaction with WRN in Saos-2 cell extracts, while an anti-BRCA1 immunoprecipitation from AG11395 WS cell extracts and an anti-IgG immunoprecipitation from Saos-2 cell extracts serve as negative controls. Immunoprecipitation input lanes (10% of the total extract) of WRN and BRCA1 are shown in the left panels. **B**. VA-13 cells were synchronized with aphidicolin and collected at various time points post-release from aphidicolin arrest. Cell cycle distribution was analyzed by flow cytometry at 2, 6 or 12 hours post-release as depicted in the histograms. **C**. Synchronized nuclear extracts were immunoprecipitated with antibodies to WRN and the immunoprecipitated proteins subjected to western blotting with antibodies to WRN (top) or BRCA1 (bottom). Immunoprecipitation with anti-BLM serves as a positive control for the interaction with WRN and BRCA1 in VA-13 cell extracts, while an anti-WRN immunoprecipitation from AG11395 WS cell extracts serves as a negative control.

**Figure 7:**
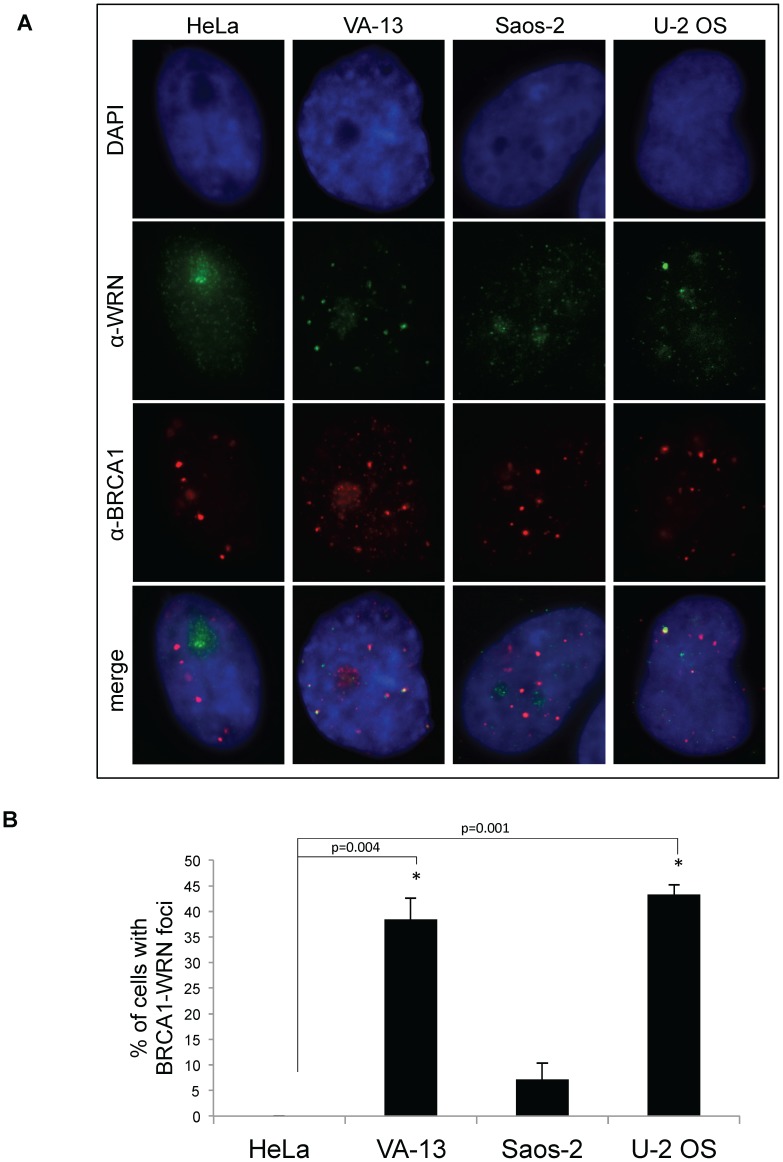
Immunofluorescence of WRN and BRCA1 in telomerase-positive and ALT cells. **A**. Representative micrograph images of cells from the telomerase-positive cell line HeLa and three ALT cell lines, VA-13, Saos-2 and U-2 OS. Cells were fixed and immunofluorescently labeled with antibodies to BRCA1 (red) and WRN (green), and the nucleus is stained with DAPI (blue). **B**. A graphical representation of at least three independent experiments averaged to yield the percentage of each cell type with co-localized BRCA1-WRN foci.

**Figure 8:**
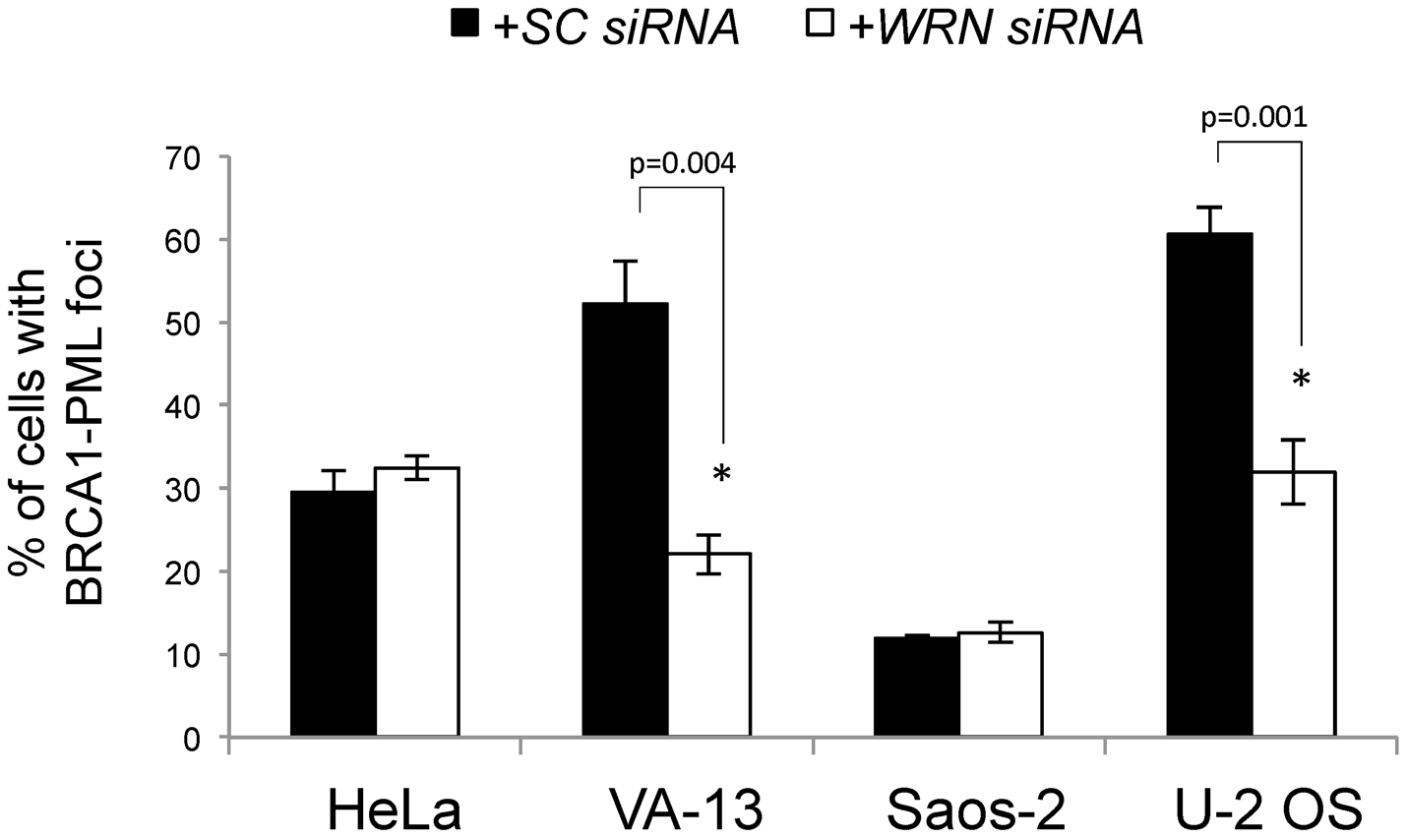
BRCA1 localization to PML bodies after *WRN* knockdown. Telomerase-positive HeLa cells and three ALT cell lines, VA-13, Saos-2 and U-2 OS, were transfected with SC or *WRN siRNAs* and fixed 48 hours after transfection. Fixed cells were immunofluorescently labeled with antibodies to BRCA1 and PML and scored for co-localization. Percentage of cells with co-localized foci were averaged from three independent experiments.

Finally, we tested whether protein partners of WRN correlated with the requirement for WRN in ALT cell lines. The BRCA1 tumor suppressor, germline mutations in which predispose to ovarian and breast cancer, localizes to APBs [42]. BLM interacts with BRCA1 in the BRCA1-associated genome surveillance (BASC) complex to sense and repair DNA damage [43]; WRN interacts with BRCA1 specifically in response to interstrand crosslinks [44]. As this evidence suggests that WRN and BRCA1 may associate at telomeres, ALT cells were examined for an interaction between WRN and BRCA1. Immunoprecipitation-western experiments with anti-BRCA1 and anti-WRN antibodies revealed a robust interaction between WRN and BRCA1 in VA-13 ALT cells, but not in Saos-2 (**Fig 6A**). Reversing the antibodies for immunoprecipitations generated similar results (data not shown). This interaction was most prominent in cells synchronized in G_2_/M-phases of the cell cycle when ALT is thought to occur (**Fig 6B, 6C**). Immunofluorescent staining demonstrated co-localization of WRN and BRCA1 foci in asynchronous fixed VA-13 and U-2 OS cells (**Fig 7A**). Quantification of the percentage of cells with co-localized foci demonstrated that 38% of VA-13 (*p* = 0.004), 43% of U-2 OS (*p* = 0.001), and 7% of Saos-2 (*p* = 0.061) cells demonstrate co-localization of WRN and BRCA1 as compared to the lack of co-localization in HeLa cells (**Fig 7B**). Furthermore, transfection of *WRN siRNAs* decreased BRCA1 localization to APBs 48 hours after knockdown in VA-13 (*p* = 0.004) and U-2 OS (*p* = 0.001), but not in Saos-2 cells (*p* = 0.376) (**Fig 8**). BRCA1 co-localization to PML bodies, as previously reported in telomerase-positive HeLa cells [45], was unaffected by *WRN siRNA* transfection (*p* = 0.183) (**Fig 8**). Taken with the results on APB formation (**Fig 3**), these results are consistent with WRN functions in APB assembly in VA-13 and U-2 OS cells.

### Evidence for an ALT mechanism switch in U-2 OS cells transfected with p53 and WRN siRNAs

**Figure 9:**
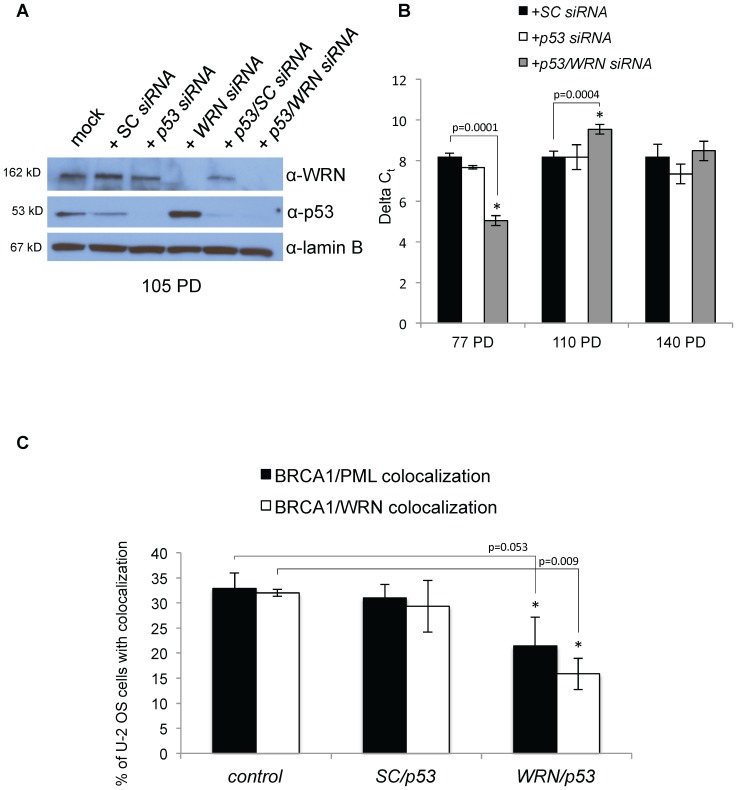
U-2 OS cells undergo an ALT mechanism switch under prolonged *WRN/p53* knockdown. **A**. Pooled *WRN siRNAs*, *p53 siRNAs*, and/or scrambled control (SC) *siRNAs* were transfected into U-2 OS ALT cells and whole cell extracts were collected 48 hours after transfection. Each lysate was separated by SDS-PAGE and western blotted with antibodies to WRN (top), p53 (middle), and lamin B (as a loading control, bottom). **B**. The difference in the cycle threshold (C_t_) between a telomere-specific PCR reaction and a single copy gene (*ALB*) PCR reaction is calculated for each sample as the ΔC_t_, which represents the average relative telomere repeat length. Telomere lengths of U-2 OS cells were measured following transfection of SC, *p53* or *p53*/*WRN siRNAs* at 77, 110 or 140 PD. **C**. BRCA1 localization to PML bodies and co-localization with WRN were observed after cessation of *siRNA* transfection after 140 PD of *SC/p53* or *WRN/p53* knockdown. Cells were fixed and immunofluorescently labeled with antibodies to BRCA1 and WRN or PML. Percentage of cells with co-localized foci was averaged from three independent experiments. (ALT = alternative lengthening of telomeres; PD = population doubling; TRAP = telomere repeat amplification protocol)

Shortened telomere lengths were observed in U-2 OS cells following continuous *WRN/p53* knockdown at 77 PD (**Fig 2**). Cells were maintained in culture with *siRNA* transfections and further changes in telomere length evaluated. No changes in cell viability were observed although similar knockdown efficiencies were observed at later population doublings (105 PD) (**Fig 9A**). Therefore, telomere lengths were again measured using qRT-PCR at 110 PD and 140 PD. Surprisingly, telomeres had elongated in cells continuously transfected with *WRN* and *p53 siRNAs* to lengths consistent with control transfected cells (**Fig 9B**). The TRAP assay was used to determine whether telomerase reactivation had elongated telomeres as observed in Ballal *et al.* 2009 [31]. TRAP assays using extracts from transfected U-2 OS cells were still negative, showing that telomerase did not elongate telomeres (**data not shown**). This result suggests that an alternative ALT mechanism that does not require WRN was activated in U-2 OS ALT cells. APBs were present at levels comparable to those observed prior to telomere shortening/lengthening events (data not shown), and C-circle assays demonstrated no significant difference in C-circles in these cells (data not shown). In contrast to APBs and C-circles, co-localization of BRCA1 with WRN (*p* = 0.009) and BRCA1 localization to PML bodies (*p* = 0.05) were altered **(** **Fig 9C**) in these late-generation U-2 OS knockdown cells, similar to levels in Saos-2 ALT cells in which WRN knockdown has no effect.

### WRN loss increases, but BRCA1 loss reduces, telomere recombination

**Figure 10:**
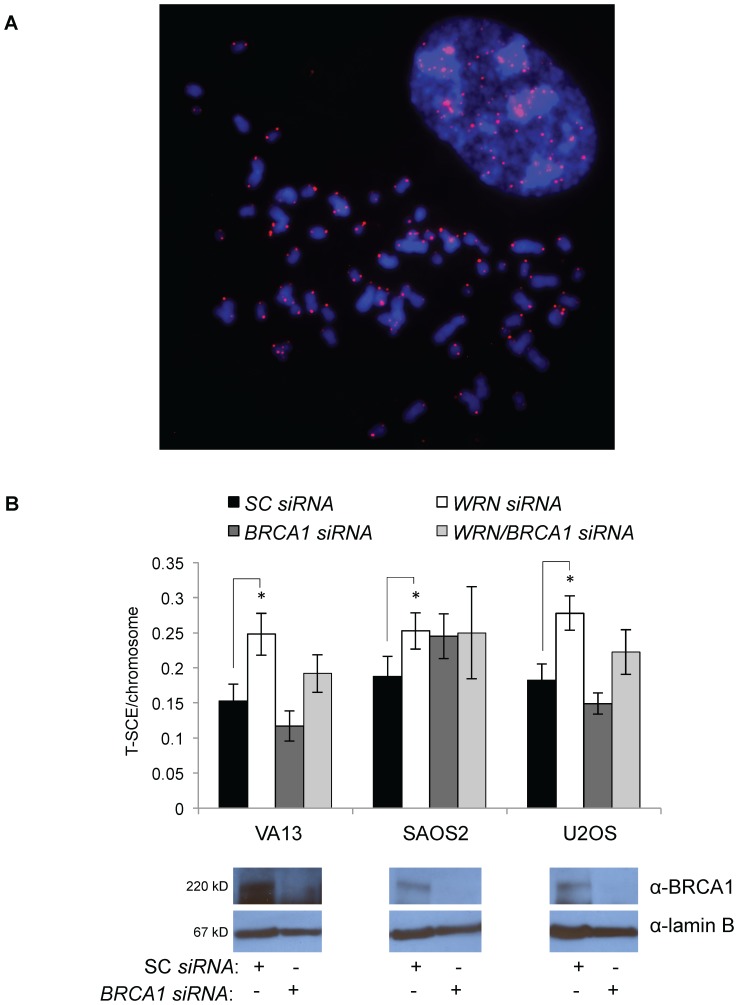
Telomere recombination measured by T-SCE is variably affected by *WRN* and/or *BRCA1* knockdown. ALT cell lines VA-13, Saos-2, and U-2 OS were transfected with *WRN*, *BRCA1*, or *WRN/BRCA1 siRNAs* and metaphase spreads were processed for T-SCE after 72 hours. Telomeres were fluorescently labeled and analyzed. **A**. A representative image of a metaphase spread from VA-13 cells shows DAPI-labeled chromosomes in blue and Cy-3 labeled telomeres in red. **B**. Results depict the frequency of T-SCE per chromosome analyzed from at least 30 metaphase spreads per treatment group. Western blots depict the ability of *BRCA1 siRNAs* to knock down BRCA1 in each cell line.

WRN represses telomere sister chromatid exchange (T-SCE), a measure of telomere recombination, presumably by preventing aberrant recombination between sister chromatids [17], [53]. BRCA1 is extensively involved in homologous recombination, although its role specifically in telomere recombination remains undefined. To determine the role of WRN in alternative telomere maintenance, we measured T-SCE after transfecting ALT cells with non-targeting or *WRN siRNAs* (**Fig 10A**). We also transfected cells with *BRCA1 siRNAs* or *WRN* and *BRCA1 siRNAs* to determine whether these proteins may be functioning together to maintain telomeres in ALT cells. In VA-13, Saos-2, and U-2 OS cells, *WRN* knockdown significantly increased the frequency of T-SCE per chromosome after 72 hours (**Fig 10B**). *BRCA1* knockdown only modestly impacted telomere recombination after 72 hours—T-SCE decreased in VA-13 and U-2 OS cells and slightly increased in Saos-2 cells. Knockdown of both *WRN* and *BRCA1* had an intermediate effect on T-SCE rates in VA-13 and U-2 OS cells, but affected T-SCE similarly to only *BRCA1* or *WRN* knockdown in Saos-2 cells.

## Discussion

The ALT pathway maintains telomeres in the absence of telomerase, but its precise mechanism or mechanisms are largely unknown. A small number of proteins has proven to be essential for ALT. Loss of MRN complex components [33], [34], SMC5/6 [32] or the BLM helicase [23] inhibits ALT and result in telomere shortening. Loss of other ALT-related proteins do not shorten telomeres but lead to telomere dysfunction [46]. Here, we show that the WRN helicase, germline mutations in which lead to WS, is required for alternative telomere maintenance in some, but not all, ALT cell lines. Our results suggest that there are at least two different telomerase-independent telomere maintenance mechanisms. These results are consistent with previous studies that have identified varied responses to *WRN* knockdown by different cell lines [51].

Previous studies have also suggested a function for WRN at the telomere through its interactions with telomeric proteins and structures mimicking telomeric DNA. Our results reveal that WRN is necessary for telomere maintenance in VA-13 and U-2 OS cells and is associated with APB formation, as impaired APB formation occurs with telomere shortening following *WRN* knockdown. The AG11395 immortalized WS cell line that maintains telomeres by ALT also lacks APBs [2]. Expression of WRN in AG11395 cells results in APB formation, a gain of telomere sequences and the conversion of type I-like telomeres to type II-like telomeres [24]. APBs are a site of BrdU incorporation [7], suggesting that some ALT cells may use APB-localized proteins for telomere elongation. DNA recombination functions of WRN [47], [48] could support a model of similar function in recombination-mediated telomere elongation within APBs. Previous studies have also demonstrated that suppression of APBs correlates with telomere shortening [33]. Other cell lines, such as Saos-2, may not require WRN either for the formation or function of APBs. Saos-2 cells have high C-circle levels compared to other types of ALT cells [35]. It is plausible that Saos-2 cells rely on ECTR instead of APBs for ALT. C-circle experiments in our studies suggest that WRN is not involved in the formation/degradation of ECTR and that ECTR levels do not directly account for telomere length changes observed in VA-13 and U-2 OS cells.

ALT-specific requirements for WRN in VA-13 and U-2 OS cells correlate with an interaction of WRN and BRCA1, also associated with a variety of other cellular functions. WRN interacts with BRCA1 in response to interstrand crosslinks; BRCA1 can stimulate both WRN exonuclease and helicase activity *in vitro* [44]; WRN interacts with BRCA1 at telomeres of U-2 OS ALT cells in response to resveratrol treatment [49]. BRCA1 is also implicated in telomere maintenance as BRCA1 deficiency results in telomere loss and telomere dysfunction in T-cells [50]. Here, we confirm an ALT-specific interaction between WRN and BRCA1 in VA-13 and U-2 OS ALT cells and speculate that this interaction facilitates one type of telomere maintenance in the absence of telomerase.

Although previous studies have shown that U-2 OS ALT cells are resistant to *WRN siRNA* knockdown [51], we observed the induction of apoptosis 17 PD after initial *siRNA* transfection. These differences are likely due to the plasticity of telomere maintenance mechanisms in U-2 OS cells. This is further strengthened by our observation that long-term knockdown of *WRN* in U-2 OS ALT cells, in combination with *p53* to prevent cell death, induces a presumed ALT mechanism switch. Telomerase was not activated in these cells, although they experienced a robust shortening and subsequent telomere lengthening in the absence of WRN. Initial telomere shortening was observed by 77 PD accompanied by a loss of APBs, suggesting that WRN was required for ALT. By 110 PD, U-2 OS telomeres lengthened in the absence of WRN, demonstrating that WRN was no longer required for ALT. We believe this change represents a switch from one ALT mechanism to another. No change in APBs levels or ECTR levels was noted between these cells, although noted differences included the localization of BRCA1 to PML bodies and co-localization of BRCA1 with WRN. Further work will characterize these ALT mechanisms.

The ability of WRN to prevent telomere loss in VA-13 and U-2 OS ALT cells may be accomplished by suppressing aberrant recombination within telomeric tracts. T-SCE are frequent in ALT cells [52] and increase after WRN knockdown in telomerase-negative human fibroblasts [53], implicating RecQ-like helicases in the suppression of aberrant recombination in ALT. We measured significantly increased telomere recombination after *WRN* knockdown, while *BRCA1* knockdown reduced recombination. Similar changes in T-SCE frequency were observed in VA-13 and U-2 OS cells, while Saos-2 cells slightly increased telomere recombination rates in response to *WRN* and/or *BRCA1* loss. While these results still do not illuminate the precise mechanisms of alternative telomere maintenance in each cell line, they demonstrate that Saos-2 cells fundamentally differ from VA-13 and U-2 OS cells. It seems clear that there are major mechanistic differences within the general ALT classification.

Additional studies have demonstrated an increase in ECTR in the absence of WRN, supporting a WRN function in preventing aberrant telomere recombination [54]. Recombination within telomere tracts can result in genomic instability and telomere dysfunction, as following the loss of POT1 [55]. Although we did not detect a change in ECTR following WRN knockdown, the C-circle assay may be limited in its ability to detect small changes in C-circle levels. WRN functions to resolve telomeric D-loops *in vitro* [11] and resolve G4 structures [56] assumed to arise within the G-rich telomeric DNA and interrupt DNA metabolism. Loss of WRN may promote the stalling of DNA replication machinery within the G-rich telomere strand, increasing recombination within the telomere to bypass the stalled region.

Data from our laboratory suggests very different roles for BLM and WRN in ALT. A recent study by Mendez-Bermudez *et al*. [57] further supports the segregation of ALT-specific BLM and WRN functions, despite similarities in protein function and specificity. Telomere mutations are absent in WS ALT cells, suggesting that WRN may facilitate complex telomere mutations that arise by interactions between non-homologous telomeric sequences. These data are concordant with the notion that WRN is involved in non-homologous DNA interactions, while BLM primarily functions in homologous interactions. Further, loss of BLM in WS ALT cells increases telomeric mutation frequency [57], suggesting BLM and WRN function in different pathways that both promote telomeric recombination.

We have shown that the WRN helicase is required to maintain telomeres in the absence of telomerase in some, but not all, immortalized human ALT cell lines. The variable WRN requirement suggests multiple ALT mechanisms, which may reflect differential use of templates for telomere recombination, including preferences for ECTR, homologous chromosomes or sister chromatids. These varied *WRN* knockdown results are consistent with previous studies that have demonstrated cell-specific responses to WRN loss, including normal growth in U-2 OS ALT cells and cell death/senescence in HIO 117 ALT cells [51]. The requirement for WRN in ALT correlates with an interaction between WRN and BRCA1, suggesting ALT mechanisms may be classified by unique protein interactions. Finally, our work argues for a variable requirement for DNA repair proteins in ALT mechanisms and a plasticity of these mechanisms. Taken together, these results suggest different ALT cell lines use unique mechanisms of telomere elongation and imply that ALT is a composite of telomerase-independent mechanisms. Further work is required to understand how these mechanisms differ mechanistically and phenotypically. Importantly, understanding the differences within ALT will allow better classification of ALT cells and may reconcile inconsistent results across different cell lines.

Telomere maintenance mechanisms are essential for cellular immortalization of tumors. While the majority of tumors activate telomerase, a significant proportion uses ALT—implicating ALT mechanisms as attractive therapeutic targets. Our studies have identified different ALT mechanisms and identified tumors that are mosaic for cells expressing telomerase and cells with characteristics of ALT [58]. The presence of ALT makes telomerase-based therapeutics ineffective in those mosaic tumors. These studies open avenues for utilizing combinatorial therapeutics targeting each mechanism and necessitate further understanding of ALT and classification of tumors with respect to telomere maintenance mechanisms.

## Materials and Methods

### Cell lines

Immortalized human cell lines WI-38 VA-13/2RA, Saos-2, U-2 OS, HeLa and MCF7 were obtained from ATCC. Saos-2 and U-2 OS are ALT cell lines derived from osteosarcomas; WI-38 VA-13/2RA is an ALT cell line derived from lung fibroblasts; HeLa is a telomerase-positive cell line derived from a cervical adenocarcinoma; and MCF7 is a telomerase-positive cell line derived from a breast adenocarcinoma. Saos-2, HeLa and MCF7 cells were grown in Dulbecco's Modified Eagle Medium (Invitrogen); WI-38 VA-13/2RA cells were grown in Minimal Essential Medium (Invitrogen); and U-2 OS cells were grown in McCoy's 5A Medium (Invitrogen). All cells were grown in media containing 10% fetal bovine serum (HyClone) and maintained at 37°C with 5% CO_2_. Identity of all cells lines used was confirmed by short tandem repeat (STR) profiling using the Cell ID System (Promega). Allelic profiles for 10 STR loci (*D21S11, TH01, TPOX, vWA, Amelogenin, CSF1PO, D16S539, D7S820, D13S317* and *D5S818*) were analyzed and confirmed (**Table 1**) in both control cells and after long-term *siRNA* knockdown.

**Table 1:** STR profiling of the indicated loci to confirm cell line identities.

	*Amelogenin*	*CSF1PO*	*D13S317*	*D16S539*	*D5S818*	*D7S820*	*THO1*	*TPOX*	*vWA*
HeLa	X	9, 10	12, 13.3	9, 10	11, 12	8, 12	7	8, 12	16, 18
MCF7	X	10	11	11, 12	11, 12	8, 9	6	9, 12	14, 15
VA-13	X	10, 12	11	11, 12	10	9, 11	9.3	8	19, 20
Saos-2	X	10	12, 13	12, 13	12	8, 10	4, 6, 9	8	18
U-2 OS	X	13	13	11, 12	11	11, 12	6, 9.3	11, 12	14, 18

### siRNA knockdown

ON-Target Plus pooled *siRNAs* targeting *WRN* (Dharmacon) were transfected into cultured cells in triplicate wells using Dharmafect 1 transfection reagent (Dharmacon) per manufacturer's directions at a final concentration of 50 nM. Cells were passaged into fresh media containing *siRNAs* every 5 days. *TRF2 siRNA* (Santa Cruz) and pooled *p53 siRNAs* (Dharmacon) were transfected at 50 nM as above. Pooled scrambled sequence control *siRNAs* (Dharmacon) are non-targeting and were transfected at 50 nM as above. *BRCA1 siRNAs* represent a combination of predesigned pooled *siRNAs* (Dharmacon) and custom *siRNAs* designed to the 3′UTR [59] and were transfected as above at 50 nM each. *BLM siRNA* (Ambion) was transfected at 40 nM using Lipofectamine 2000 transfection reagent (Invitrogen) per manufacturer's directions.

### Western blot

Western blotting of whole cell extracts was performed according to standard procedures. Primary antibodies used were: anti-WRN (Abcam), anti-BLM (Bethyl Laboratories), anti-lamin B (Santa Cruz), anti-TRF2 (Imgenex), anti-p53 (Santa Cruz), anti-PARP1 p85 fragment (Promega), and anti-BRCA1 [60]. Secondary antibodies used were: horseradish-peroxidase conjugated goat-anti-mouse, goat-anti-rabbit and rabbit-anti-goat antibodies (Jackson ImmunoResearch Laboratories).

### qRT-PCR assay

DNA extracts were collected from cells using the Genomic DNA Isolation kit (Trevigen). Telomeric DNA was amplified from 30 ng total genomic DNA using Sybr green PCR master mix (Applied Biosystems) as previously described [29]. *ALB* was amplified as a single copy gene control. Both PCR reactions were performed in triplicate wells on an Applied Biosystems 7900HT Fast-Real Time PCR machine. Amplified products were visualized on a 4% agarose gel to verify expected products. For verification of telomere length analysis, TRF Southern blots were performed using standard procedures.

### TRAP assay

Telomerase activity was measured using the TRAPeze kit (Millipore) per manufacturer's directions. To prevent interference by PCR inhibitors in the cell extracts, samples were phenol-chloroform extracted after template elongation and before PCR amplification. Products were separated on 10% SDS-PAGE gels and dried on a BioRad Model 583 gel dryer. Gels were exposed to phosphor screens (GE) overnight and imaged on a Typhoon 9410 variable mode imager (GE). Negative control samples include no cell extract, while positive controls represent a manufacturer-provided telomerase-positive cell extract.

### C-circle assay

Total DNA was extracted from cells using the DNeasy kit (Qiagen) and analyzed for C-circles as previously described [35]. Because C-circle levels vary in different ALT cell lines, 100 ng of DNA was used for Saos-2 and U-2 OS cells, and 1000 ng for VA-13 cells, HeLa cells and MCF7 cells. To control for DNA input, one sample was undigested and not subjected to the C-circle assay (total telomere DNA), while another sample was digested with *Hinf*I*/Rsa*I restriction enzymes and *Exo*I*/Exo*V*/Exoλ* exonucleases, and subjected to the C-circle assay. C96 is a synthetic C-circle that serves as a positive control [35]. Products were separated on a 0.8% agarose gel and transferred to a nylon charged membrane (Hybond). Telomeric products were visualized by hybridization with a ^32^P-end labeled (CCCTAA)_3_ probe at 42°C in UltraHyb-Oligo hybridization buffer (Ambion). Membranes were washed and exposed to a phosphor screen (GE) overnight and imaged on a Typhoon 9410 variable mode imager (GE).

### Immunofluorescence

Cells were grown on coverslips (Fisher), washed in PBS and fixed in 4% paraformaldehyde. Cells were permeabilized in 0.25% Triton-x-100 in PBS and washed before blocking in 10% normal goat serum (Sigma) and subsequent staining with the indicated antibodies in 1% BSA/0.1% Tween/PBS: anti-TRF2 (Imgenex), anti-PML (Abcam), anti-γH2AX (Millipore), anti-WRN (Active Motif), or anti-BRCA1 (Calbiochem). Primary antibodies were visualized with fluorescent AlexaFluor (Invitrogen) secondary antibodies. For FISH, cells were dehydrated after fixation in an ethanol series, rehydrated in 2X SSC washes and hybridized with a Cy-3 conjugated PNA telomeric probe (Panagene) at 67 μg/mL in 70% formamide/2X SSC. Cells were washed in 0.1% Tween/PBS at 55°C followed by a rinse in 0.1% Tween/2X SSC at room temperature. Coverslips were then rinsed, air-dried and mounted with VectaShield DAPI mounting medium (Vector Labs) onto glass slides (Fisher). Cells were imaged using a Zeiss AxioVert 200 M with an attached AxioCam MRm camera or an Olympus FV 1000 spectral confocal system. Fifty cells in each of three independent experiments were analyzed blind for co-localization.

### Immunoprecipitation

Nuclear extracts were prepared from cultured cells as previously described [61]. Two milligrams of extract were immunoprecipitated with antibodies to BRCA1 (Santa Cruz) or WRN (Abcam) using Protein A/G agarose beads (Santa Cruz) and proteins were separated by SDS-PAGE and blotted onto a PVDF membrane (Millipore). Western blotting was performed as described previously. For synchronization, cells were arrested with 1 ug/mL aphidicolin for 24 hours and extracts were collected 12 hours after release from aphidicolin treatment. Cell cycle profiles were confirmed by flow cytometry of propidium iodide-stained cells.

### T-SCE

Cells were transfected with pooled scrambled sequence control *siRNAs* or *WRN siRNAs* as above. Pooled *BRCA1 siRNAs* (Dharmacon) were transfected at 50 nM with Dharmafect 1, then reverse transfected again 24 hours later. Cells were treated and metaphase spreads prepared 72 hours after the initial transfection and stained as previously described [53]. At least 30 metaphase spreads per treatment were analyzed.

### Statistical analysis

The two-sample t-test was used to analyze significance by comparing each treatment group to the control for each cell line with an assumption of unequal variances.

## Supporting Information

Figure S1 **Persistance of** ***WRN siRNA*** **knockdown in HeLa cells.** Pooled *WRN siRNAs* or scrambled control (SC) *siRNAs* were transfected into HeLa cells and whole cell extracts were collected 4, 5 or 6 days after transfection. Lysates were separated by SDS-PAGE and western blotted with antibodies to WRN (top) and lamin B (as a loading control, bottom). Similar results were observed with additional cell lines (data not shown).
(TIF)

Figure S2 **Correlation between TRF Southern blot and qRT-PCR telomere length analyses.** Five tumor DNA samples were separately collected [58] and telomere lengths measured from the same samples by both TRF Southern blot (left) and qRT-PCR (right). HeLa and WI38 VA-13/2RA are positive controls for telomerase and ALT cells, respectively; no DNA and lambda DNA represent negative controls. Relative telomere length represents the difference between Ct values for a telomere PCR reaction and a single copy gene (albumin) PCR reaction.
(TIF)

Figure S3 **TRAP assay of cell extracts following transfection of SC or** ***WRN siRNAs*** **.** Cell extracts were assayed for telomerase activity by the TRAP assay. IC represents the internal control band for the assay, with the laddered bands representing TRAP products. HeLa (lanes 1–4) and MCF7 cells (lanes 5–8) are telomerase-positive cell lines and depict clear laddering, while Saos-2 (lanes 9–12), VA-13 (lanes 13–16) and U-2 OS (lanes 19–30) are ALT cells and lack telomerase activity. A heat denatured negative control is included for each cell type. In addition, manufacturer-provided negative (lane 17, lane 31) and positive (lane 18, lane 32) TRAP kit controls are included on the far right of the gels. (TRAP = telomere repeat amplification protocol).
(TIF)

Figure S4 **C-circle assay of ALT cell lines before and after transfection with SC or** ***WRN siRNAs*** **.** Total DNA extracted from HeLa, Saos-2, VA-13 and U-2 OS cell lines at 20 PD was examined for ECTR by the C-circle assay. Total telomeric DNA is shown in the bottom panel as an input control. Only C-circle products are visualized in the top panel after linear DNA is digested and subjected to the C-circle assay. Telomeric products are visualized with a ^32^P-end labeled (CCCTAA)_3_ probe. C96 is a synthetic C-circle and serves as a positive control for the assay, while DNA from telomerase-positive HeLa cells serves as a negative control.
(TIF)

## References

[1] Lundblad V, Blackburn EH (1993) An alternative pathway for yeast telomere maintenance rescues est1^−^ senescence. Cell 73: 347–360. 8477448 10.1016/0092-8674(93)90234-h

[2] Fasching CL, Bower K, Reddel RR (2005) Telomerase-independent telomere length maintenance in the absence of alternative lengthening of telomeres-associated promyelocytic leukemia bodies. Cancer Res 65: 2722–2729. 15805271 10.1158/0008-5472.CAN-04-2881

[3] Marciniak RA, Cavazos D, Montellano R, Chen Q, Guarente L, et al (2005) A novel telomere structure in a human alternative lengthening of telomere cell line. Cancer Res 65: 2730–2737. 15805272 10.1158/0008-5472.CAN-04-2888

[4] Dunham MA, Neumann AA, Fasching CL, Reddel RR (2000) Telomere maintenance by recombination in human cells. Nat Genet 26: 447–450. 11101843 10.1038/82586

[5] Muntoni A, Neumann AA, Hills M, Reddel RR (2008) Telomere elongation involves intra-molecular DNA replication in cells utilizing alternative lengthening of telomeres. Hum Mol Gen 18: 1017–1027. 19095716 10.1093/hmg/ddn436 PMC2649016

[6] Yeager TR, Neumann AA, Englezou A, Huschtscha LI, Noble JR, et al (1999) Telomerase-negative immortalized human cells contain a novel type of promyelocytic leukemia (PML) body. Cancer Res 59: 4175–4179. 10485449

[7] Wu G, Lee WH, Chen PL (2000) NBS1 and TRF1 colocalize at promyelocytic leukemia bodies during late S/G_2_ phases in immortalized telomerase-negative cells. J Biol Chem 275: 30618–30622. 10913111 10.1074/jbc.C000390200

[8] Yankiwski V, Marciniak RA, Guarente L, Neff NF (2000) Nuclear structure in normal and Bloom syndrome cells. Proc Natl Acad Sci USA 97: 5214–5219. 10779560 10.1073/pnas.090525897 PMC25808

[9] Johnson FB, Marciniak RA, McVey M, Stewart SA, Hahn WC, et al (2001) The Saccharomyces cerevisiae WRN homolog Sgs1p participates in telomere maintenance in cells lacking telomerase. EMBO J 20: 905–913. 11179234 10.1093/emboj/20.4.905 PMC145415

[10] Lillard-Wetherell K, Machwe A, Langland GT, Combs KA, Behbehani GK, et al (2004) Association and regulation of the BLM helicase by the telomere proteins TRF1 and TRF2. Hum Mol Genet 13: 1919–1932. 15229185 10.1093/hmg/ddh193

[11] Opresko PL, Otterlei M, Graakjaer J, Bruheim P, Dawut L, et al (2004) The Werner syndrome helicase and exonuclease cooperate to resolve telomeric D loops in a manner regulated by TRF1 and TRF2. Mol Cell 14: 763–774. 15200954 10.1016/j.molcel.2004.05.023

[12] Stavropoulos DJ, Bradshaw PS, Li X, Pasic I, Truong K, et al (2002) The Bloom syndrome helicase BLM interacts with TRF2 in ALT cells and promotes telomeric DNA synthesis. Hum Mol Genet 11: 3135–3144. 12444098 10.1093/hmg/11.25.3135

[13] Opresko PL, von Kobbe C, Laine JP, Harrigan J, Hickson ID, et al (2002) Telomere-binding protein TRF2 binds to and stimulates the Werner and Bloom syndrome helicases. J Biol Chem 277: 41110–41119. 12181313 10.1074/jbc.M205396200

[14] Opresko PL, Mason PA, Podell ER, Lei M, Hickson ID, et al (2005) POT1 stimulates RecQ helicases WRN and BLM to unwind telomeric DNA substrates. J Biol Chem 280: 32069–32080. 16030011 10.1074/jbc.M505211200

[15] Schulz VP, Zakian VA, Ogburn CE, McKay J, Jarzebowicz AA, et al (1996) Accelerated loss of telomeric repeats may not explain accelerated replicative decline of Werner syndrome cells. Hum Genet 97: 750–754. 8641691 10.1007/BF02346184

[16] Tahara H, Tokutake Y, Maeda S, Kataoka H, Watanabe T, et al (1997) Abnormal telomere dynamics of B-lymphoblastoid cell strains from Werner's syndrome patients transformed by Epstein-Barr virus. Oncogene 15: 1911–1920. 9365237 10.1038/sj.onc.1201377

[17] Laud PR, Multani AS, Bailey SM, Wu L, Ma J, et al (2005) Elevated telomere-telomere recombination in WRN-deficient, telomere dysfunctional cells promotes escape from senescence and engagement of the ALT pathway. Genes Dev 19: 2560–2570. 16264192 10.1101/gad.1321305 PMC1276730

[18] Crabbe L, Verdun RE, Haggblom CI, Karlseder J (2004) Defective telomere lagging strand synthesis in cells lacking WRN helicase activity. Science 306: 1951–1953. 15591207 10.1126/science.1103619

[19] Wyllie FS, Jones CJ, Skinner JW, Haughton MF, Wallis C, et al (2000) Telomerase prevents the accelerated cell ageing of Werner syndrome fibroblasts. Nat Genet 24: 16–17. 10615119 10.1038/71630

[20] Lombard DB, Beard C, Johnson B, Marciniak RA, Dausman J, et al (2000) Mutations in the Wrn gene in mice accelerate mortality in a p53-null background. Mol Cell Biol 20: 3286–3291. 10757812 10.1128/mcb.20.9.3286-3291.2000 PMC85622

[21] Chang S, Multani AS, Cabrera NG, Naylor ML, Laud P, et al (2004) Essential role of limiting telomeres in the pathogenesis of Werner syndrome. Nat Genet 36: 877–882. 15235603 10.1038/ng1389

[22] Du X, Shen J, Kugan N, Furth EE, Lombard DB, et al (2004) Telomere shortening exposes functions for the mouse Werner and Bloom syndrome genes. Mol Cell Biol 24: 8437–8446. 15367665 10.1128/MCB.24.19.8437-8446.2004 PMC516757

[23] Bhattacharyya S, Keirsey J, Russell B, Kavecansky J, Lillard-Wetherell K, et al (2009) Telomerase-associated protein 1, HSP90, and topoisomerase IIá associate directly with the BLM helicase in immortalized cells using ALT and modulate its helicase activity using telomeric DNA substrates. J Biol Chem 284: 14966–14977. 19329795 10.1074/jbc.M900195200 PMC2685679

[24] Siddiqa A, Cavazos D, Chavez J, Long L, Marciniak RA (2012) Modulation of telomeres in alternative lengthening of telomeres type I-like human cells by the expression of Werner protein and telomerase. J Oncol 806382: doi: 10.1155/2012/806382 10.1155/2012/806382 PMC3321466 22545052

[25] Harley CB, Futcher AB, Greider CW (1990) Telomeres shorten during ageing of human fibroblasts. Nature 345: 458–460. 2342578 10.1038/345458a0

[26] Allsopp RC, Vaziri H, Patterson C, Goldstein S, Younglai EV, et al (1992) Telomere length predicts replicative capacity of human fibroblasts. Proc. Natl Acad Sci USA 89: 10114–10118. 10.1073/pnas.89.21.10114 PMC50288 1438199

[27] Vaziri H, Schachter F, Uchida I, Wei L, Zhu X, et al (1993) Loss of telomeric DNA during aging of normal and trisomy 21 human lymphocytes. Am J Hum Genet 52: 661–667. 8460632 PMC1682068

[28] Vaziri H, Dragowska W, Allsopp RC, Thomas TE, Harley CB, et al (1994) Evidence for a mitotic clock in human hematopoietic stem cells: loss of telomeric DNA with age. Proc Natl Acad Sci USA 91: 9857–9860. 7937905 10.1073/pnas.91.21.9857 PMC44916

[29] Cawthon RM (2009) Telomere length measurement by a novel monochrome multiplex quantitative PCR method. Nucleic Acids Res 37 : doi:10.1093/nar/gkn1027. 10.1093/nar/gkn1027 PMC2647324 19129229

[30] Hu J, Hwang SS, Liesa M, Gan B, Sahin E, et al (2012) Antitelomerase therapy provokes ALT and mitochondrial adaptive mechanisms in cancer. Cell 148: 651–663. 22341440 10.1016/j.cell.2011.12.028 PMC3286017

[31] Ballal RD, Saha T, Fan S, Haddad BR, Rosen EM (2009) BRCA1 localization to the telomere and its loss from the telomere in response to DNA damage. J Biol Chem 284: 36083–36098. 19797051 10.1074/jbc.M109.025825 PMC2794724

[32] Potts PR, Yu H (2007) The SMC5/6 complex maintains telomere length in ALT cancer cells through SUMOylation of telomere-binding proteins. Nat Struct Mol Biol 14: 581–590. 17589526 10.1038/nsmb1259

[33] Jiang WQ, Zhong ZH, Henson JD, Neumann AA, Chang ACM, et al (2005) Suppression of alternative lengthening of telomeres by Sp100-mediated sequestration of the MRE11/RAD50/NBS1 complex. Mol Cell Biol 25: 2708–2721. 15767676 10.1128/MCB.25.7.2708-2721.2005 PMC1061646

[34] Zhong ZH, Jiang WQ, Cesare AJ, Neumann AA, Wadhwa R, et al (2007) Disruption of telomere maintenance by depletion of the MRE11/RAD50/NBS1 complex in cells that use alternative lengthening of telomeres. J Biol Chem 282: 29314–29322. 17693401 10.1074/jbc.M701413200

[35] Henson JD, Cao Y, Huschtscha LI, Chang AC, Au AYM, et al (2009) DNA C-circles are specific and quantifiable markers of alternative-lengthening-of-telomeres activity. Nat Biotechnol 27: 1181–1186. 19935656 10.1038/nbt.1587

[36] Li B, Reddy S, Comai L (2011) Depletion of Ku70/80 reduces the levels of extrachromosomal telomeric circles and inhibits proliferation of ALT cells. Aging 3: 395–406. 21512205 10.18632/aging.100308 PMC3117455

[37] Takai H, Smogorzewska A, de Lange T (2003) DNA damage foci at dysfunctional telomeres. Curr Biol 13: 1549–1556. 12956959 10.1016/s0960-9822(03)00542-6

[38] d’Adda di Fagagna F, Reaper RM, Clay-Farrace L, Fiegler H, Carr P, et al (2003) A DNA damage checkpoint response in telomere-initiated senescence. Nature 426: 194–198. 14608368 10.1038/nature02118

[39] Kim S, Beausejour C, Davalos AR, Kaminker P, Heo S, et al (2004) TIN2 mediates function of TRF2 at human telomeres. J Biol Chem 279: 43799–43804. 15292264 10.1074/jbc.M408650200

[40] Hockemeyer D, Sfeir AJ, Shay JW, Wright WE, de Lange T (2005) POT1 protects telomeres from a transient DNA damage response and determines how human chromosomes end. EMBO J 24: 2667–2678. 15973431 10.1038/sj.emboj.7600733 PMC1176460

[41] Nabetani A, Yokoyama O, Ishikawa F (2004) Localization of hRAD9, hHUS1, hRAD1, and hRAD17 and caffeine-sensitive DNA replication at the alternative lengthening of telomeres-associated promyelocytic leukemia body. J Biol Chem 279: 25849–25857. 15075340 10.1074/jbc.M312652200

[42] Wu G, Jiang X, Lee WH, Chen PL (2003) Assembly of functional ALT-associated promyelocytic leukemia bodies requires Niimegen breakage syndrome 1. Cancer Res 63: 2589–2595. 12750284

[43] Wang Y, Cortez D, Yazdi P, Neff N, Elledge SJ, et al (2000) BASC, a super complex of BRCA1-associated proteins involved in the recognition and repair of aberrant DNA structures. Genes Dev 14: 927–939. 10783165 PMC316544

[44] Cheng WH, Kusumoto R, Opresko PL, Sui X, Huang S, et al (2006) Collaboration of Werner syndrome protein and BRCA1 in cellular responses to DNA interstrand cross-links. Nucleic Acids Res 34: 2751–2760. 16714450 10.1093/nar/gkl362 PMC1464112

[45] Luciani JJ, Depetris D, Usson Y, Metzler-Guillemain C, Mignon-Ravix C, et al (2006) PML nuclear bodies are highly organized DNA-protein structures with a function in heterochromatin remodeling at the G2 phase. J Cell Sci 119: 2518–2531. 16735446 10.1242/jcs.02965

[46] Gocha ARS, Harris J, Groden J (2013) Alternative mechanisms of telomere lengthening: Permissive mutations, DNA repair proteins and tumorigenic progression. Mut Res: *In press*. 10.1016/j.mrfmmm.2012.11.006 PMC3619008 23219603

[47] Saintigny Y, Makienko K, Swanson C, Emond MJ, Monnat RJ Jr (2002) Homologous recombination resolution defect in Werner syndrome. Mol Cell Biol 22: 6971–6978. 12242278 10.1128/MCB.22.20.6971-6978.2002 PMC139822

[48] Baynton K, Otterlei M, Bjoras M, von Kobbe C, Bohr VA, et al (2003) WRN interacts physically and functionally with the recombination mediator protein RAD52. J Biol Chem 278: 36476–36486. 12750383 10.1074/jbc.M303885200

[49] Rusin M, Zajkowicz A, Butkeiwicz D (2009) Resveratrol induces senescence-like growth inhibition of U-2 OS cells associated with the instability of telomeric DNA and upregulation of BRCA1. Mech Ageing Dev 130: 528–537. 19559722 10.1016/j.mad.2009.06.005

[50] McPherson JP, Hande MP, Poonepalli A, Lemmers B, Zablocki E, et al (2006) A role for BRCA1 in chromosome end maintenance. Hum Mol Genet 15: 831–838. 16446310 10.1093/hmg/ddl002

[51] Opresko PL, Calvo JP, von Kobbe C (2007) Role for the Werner syndrome protein in the promotion of tumor cell growth. Mech Ageing Dev 128: 423–436. 17624410 10.1016/j.mad.2007.05.009

[52] Bechter OE, Zou Y, Walker W, Wright WE, Shay JW (2004) Telomeric recombination in mismatch repair-deficient human colon cancer cells after telomerase inhibition. Cancer Res 64: 3444–3451. 15150096 10.1158/0008-5472.CAN-04-0323

[53] Hagelstrom RT, Blagoev KB, Niedernhofer LJ, Goodwin EH, Bailey SM (2010) Hyper-telomere recombination accelerates replicative senescence and may promote premature aging. Proc Natl Acad Sci USA 107: 15768–15773. 20798040 10.1073/pnas.1006338107 PMC2936608

[54] Li B, Jog SP, Reddy S, Comai L (2008) WRN controls formation of extrachromosomal telomeric circles and is required for TRF2^ΔB^-mediated telomere shortening. Mol Cell Biol 28: 1892–1904. 18212065 10.1128/MCB.01364-07 PMC2268394

[55] Wu L, Mutani AS, He H, Cosme-Blanco W, Deng Y, et al (2006) Pot1 deficiency initiates DNA checkpoint activation and aberrant homologous recombination at telomeres. Cell 126: 49–62. 16839876 10.1016/j.cell.2006.05.037

[56] Mohaghegh P, Karow JK, Brosh RM Jr, Bohr VA, Hickson ID (2001) The Bloom's and Werner's syndrome proteins are DNA structure-specific helicases. Nucleic Acids Res 29: 2843–2849. 11433031 10.1093/nar/29.13.2843 PMC55766

[57] Mendez-Bermudez A, Hidalgo-Bravo A, Cotton VE, Gravani A, Jeyapalan JN, et al (2012) The roles of WRN and BLM RecQ helicases in the Alternative Lenthening of Telomeres. Nucleic Acids Res 40: 10809–10820. 22989712 10.1093/nar/gks862 PMC3510502

[58] Gocha ARS, Nuovo G, Iwenofu OH, Groden J (2013) Human sarcomas are mosaic for telomerase-dependent and –independent telomere maintenance mechanisms: Implications for telomere-based therapies. Am J Pathol 182: 41–48. 23260199 10.1016/j.ajpath.2012.10.001 PMC3532709

[59] Ransburgh DJR, Chiba N, Ishioka C, Toland AE, Parvin JD (2010) Identifiction of breast cancer mutations in *BRCA1* that abolish its function in homologous DNA recombination. Cancer Res 70: 988–995. 20103620 10.1158/0008-5472.CAN-09-2850 PMC2943742

[60] Simons AM, Horwitz AA, Starita LM, Griffin K, Williams RS, et al (2006) BRCA1 DNA-binding activity is stimulated by BARD1. Cancer Res 66: 2012–2018. 16489000 10.1158/0008-5472.CAN-05-3296

[61] Jiang K, Pereira E, Maxfield M, Russell B, Goudelock DM, et al (2003) Regulation of Chk1 includes chromatin association and 14-3-3 binding following phosphorylation on Ser-345. J Biol Chem 278: 25207–25217. 12676962 10.1074/jbc.M300070200

