# Supplementary figures and images for: WRN Loss Induces Switching of Telomerase-Independent Mechanisms of Telomere Elongation
Source: PLoS One. 2014 Apr 7;9(4):e93991. doi: 10.1371/journal.pone.0093991 (PMC3977986; doi:10.1371/journal.pone.0093991)

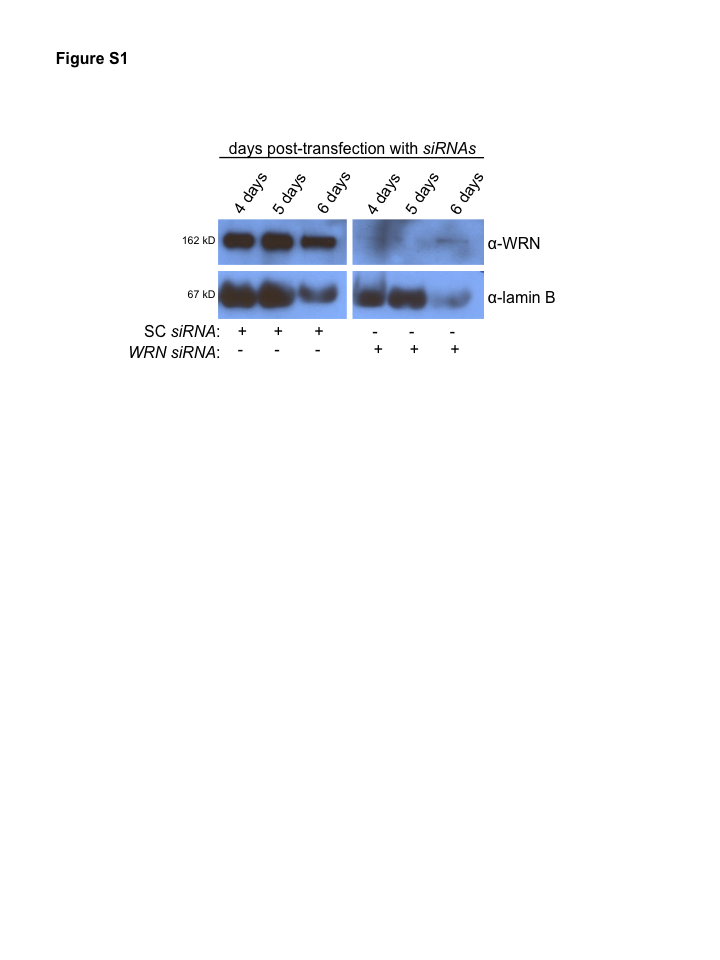

Supplement: Figure S1 — Persistance of WRN siRNA knockdown in HeLa cells. Pooled WRN siRNAs or scrambled control (SC) siRNAs were transfected into HeLa cells and whole cell extracts were collected 4, 5 or 6 days after transfection. Lysates were separated by SDS-PAGE and western blotted with antibodies to WRN (top) and lamin B (as a loading control, bottom). Similar results were observed with additional cell lines (data not shown). (TIF) [file pone.0093991.s001.tif]
